# Delayed Recognition of an Ureteropelvic Junction Obstruction in a Young Adult Female

**DOI:** 10.1155/2015/654350

**Published:** 2015-06-28

**Authors:** Ariel Schulman, Jean Paul Wuilleumier, Ervin Teper

**Affiliations:** ^1^Urology Residency Program, Maimonides Medical Center, 4802 10th Avenue, Brooklyn, NY 11219, USA; ^2^Urology Department, Maimonides Medical Center, 4802 10th Avenue, Brooklyn, NY 11219, USA

## Abstract

A percentage of ureteropelvic junction obstruction cases are clinically silent in childhood and manifest symptoms in adults. Herein we present a 25-year-old female with several years of intermittent flank pain and abdominal symptoms with prior inconclusive diagnostic workup including abdominal imaging without hydronephrosis. Ultimately, a CT scan performed during an acute pain crisis clearly identified right-sided hydronephrosis. The keys to diagnosis are awareness of this entity, a detailed history, and obtaining imaging studies during a crisis. The patient subsequently underwent a right robotic-assisted laparoscopic pyeloplasty with preservation of a lower pole crossing vessel. We highlight noteworthy features of the clinical presentation and surgical repair.

## 1. Introduction

The ureteropelvic junction is a common site of upper urinary tract obstruction commonly detected and treated in early childhood. Some cases are clinically silent through puberty and present in young adults. Due to its rare incidence in adults, vague of symptoms, and intermittent nature, appropriate diagnosis may be delayed or patient may be mistreated. We present a 25-year-old healthy female with several years of vague right flank pain and abdominal symptoms with a previously inconclusive workup by multiple physicians including advanced imaging without evidence of hydronephrosis. A CT scan performed during acute pain revealed moderate right hydronephrosis secondary to an ureteropelvic junction obstruction (UPJO) by a lower pole crossing vessel. This case emphasizes the importance of imaging at the time of symptoms to make the correct diagnosis. Furthermore, it is important to increase awareness of this phenomenon amongst our nonurologic colleagues.

## 2. Case Report

**Figure 1:**
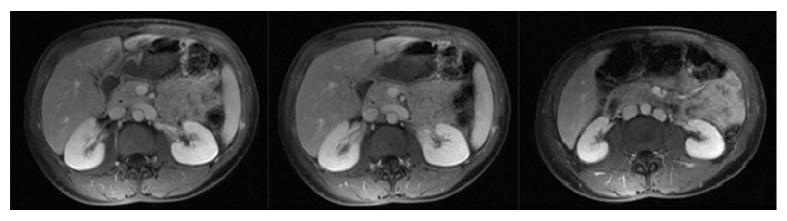
An outpatient MRI of the abdomen shows no hydronephrosis at the time of no symptoms. Incidentally noted is a duplicated right inferior vena cava distal to the renal vein.

A healthy 25-year-old female kindergarten teacher with no significant past medical history presented to the emergency department with severe, debilitating right flank pain after drinking two glasses of wine. Detailed history revealed several years of intermittent abdominal pain often precipitated by alcohol obligating her to exclude beer from her diet. Extensive previous evaluation by gastroenterology, gynecology, and Internal Medicine did not produce a conclusive diagnosis. Prior imaging included an unremarkable pelvic ultrasound and abdominal ultrasound and MRI of the abdomen without hydronephrosis (Figure 1). Past surgical history was remarkable for an appendectomy several years earlier.

**Figure 2:**
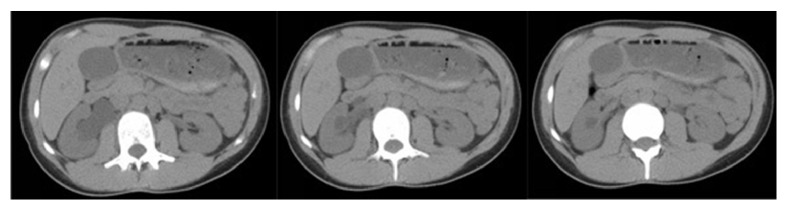
Noncontrast CT scan of the abdomen with moderate right-sided hydronephrosis proximal to an apparent lower pole renal vessel overlying the ureteropelvic junction performed at the time of acute pain.

On exam, vital signs were within normal limits and abdominal examination was unremarkable. Serum creatinine was 1.0 mg/dL. Urinalysis noted moderate leukocyte esterase, 10–25 WBCs/hpf and 2–5 RBCs/hpf with no bacteria. CT scan of the abdomen during acute pain crisis showed moderate right hydronephrosis with no hydroureter. There was no urolithiasis and renal parenchyma was preserved (Figure 2). Incidentally noted was a duplicated inferior vena cava distal to the renal veins. On detailed review, there was a lower pole vessel crossing inferior to the point of maximal hydronephrosis. The diagnosis was reviewed with the patient and she was discharged with pain medications and elective surgery was planned.

**Figure 3:**
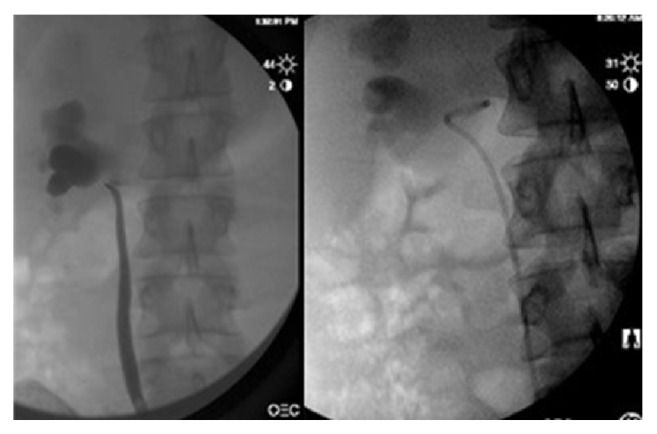
Preoperative retrograde pyelogram shows a normal caliber ureter and dilated pyelocalyceal system with an abrupt point of obstruction and “horizontal lie” of the JJ stent further suggesting the presence of a crossing vessel.

**Figure 4:**
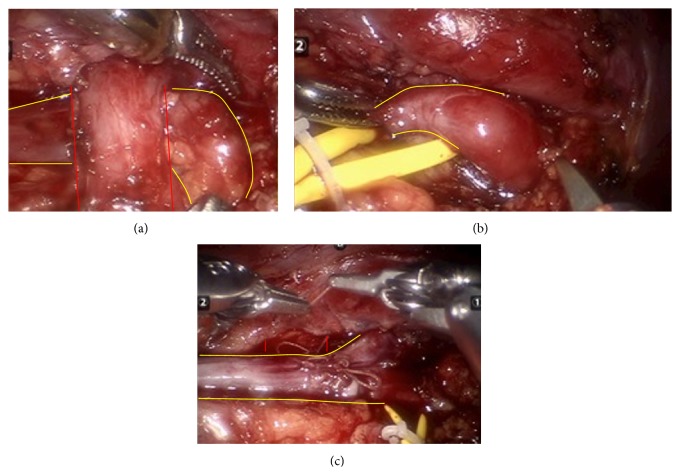
Intraoperative image (a): lower pole crossing vessel (red delineation) overlying the UPJ (yellow delineation). Intraoperative image (b): 2 cm segment of fibrotic ureter is noted in the proximal ureter and excised (yellow delineation). Intraoperative image (c): the ureter (yellow delineation) is anastomosed over a stent anterior to the preserved crossing vessel (red delineation).

Several weeks later, the patient underwent robotic-assisted laparoscopic right pyeloplasty. A preoperative retrograde pyelogram confirmed a normal caliber ureter and dilated pyelocalyceal system. The “horizontal lie” of the proximal coil of the JJ stent further suggested a crossing vessel (Figure 3). Intraoperatively, a lower pole vessel was visualized directly overlying the ureteropelvic junction. The ureter was dismembered and transposed anteriorly and a 2 cm stenotic segment of ureter was excised. The renal pelvis and ureter was reanastomosed over the stent anterior to the preserved crossing vessel (Figure 4). The patient tolerated the procedure without complications and was discharged home on postoperative day #1. The stent was removed without incident four weeks after surgery and the patient reported resolution of her symptoms.

## 3. Discussion

In 1864, Józef Dietl described intermittent violent episodes of crampy upper flank pain and nausea caused by an ureteropelvic junction obstruction defining a clinical scenario that still bears his name [1]. Chronic blockage of urine flow causes pain, urinary stasis, and renal deterioration. The most common etiologies are external compression from an aberrant vessel providing circulation of the lower pole of the kidney or intrinsic malformation of a segment of proximal ureter [2]. Less common causes include high ureteral insertion or a ureteral polyp [3].

Some cases do not become symptomatic until adulthood with pain precipitated by diuresis-inducing beverages such as coffee or alcohol. Often symptoms are vague and referred to the abdomen or pelvis [3]. Patients report intermittent bouts of pain with normal physical exam findings and imaging studies during periods of quiescence. The waxing and waning nature can lead to diagnostic confusion and there can be multiple specialist physician visits over several years without definitive diagnosis [4]. As noted in this case, the absence of hydronephrosis on abdominal imaging delays referral to urology for further evaluation.

Cross-sectional abdominal imaging at the time of acute pain is critical for identifying hydronephrosis. While a renal scan is the gold standard to document functional obstruction, a recent study by Ozayar et al. noted that almost all patients with a highly suggestive clinical picture but equivocal renal scan findings benefit from surgical correction [5]. In this case, the classic clinical history and definitive CT findings at the time of pain obviated the need for a renal scan. A nuclear scan should be performed on patients with an unclear clinical scenario or in order to establish split renal function if there are signs of parenchymal atrophy [6].

Published success of traditional open pyeloplasty is greater than 93%. Complications include bowel perforation, vascular injury, and urine leak. Most surgeons leave an indwelling ureteral stent in place for four to six weeks after surgery. If initial surgery is unsuccessful, an endopyelotomy or repeat pyeloplasty is performed [7]. In recent years minimally invasive approaches have emerged as a viable alternative with decreased pain, earlier convalescence, shorter hospital stay, and improved cosmetics. Recent studies of robotic pyeloplasty in both children and adults have produced low complication rates with long-term efficacy equivalent to an open approach [8, 9]. We perform retrograde pyelogram at the time of surgery to localize the site of obstruction for robotic port placement.

In conclusion, ureteropelvic junction obstruction is an important and fixable disease process in older children and young adults. Patients often see several physicians before a definitive diagnosis is made due to the intermittent nature and vague symptoms of this disease. Imaging at the time of symptoms is critical to document hydronephrosis. Minimally invasive techniques have shown excellent outcomes with minimal morbidity, particularly in larger children and adults.
